# Resultant moral luck and the scope of moral responsibility

**DOI:** 10.1007/s11098-023-01994-8

**Published:** 2023-06-17

**Authors:** Matthias Rolffs

**Affiliations:** Institute of Philosophy, University of Bern, Länggassstrasse 49a, 3012 Bern, Switzerland

**Keywords:** Moral responsibility, Moral luck, Degrees of responsibility, Action, Blameworthiness, Intensionality

## Abstract

Resultant moral luck occurs whenever aspects of an agent’s moral responsibility are affected by luck pertaining to the outcomes of their actions. Many authors reject the existence of moral luck in this sense, but they do so in different ways. Michael Zimmerman argues that resultant luck affects the scope of moral responsibility, but not its degree. That is, it affects what agents are responsible for, but not how responsible they are. Andrew Khoury takes a more resolute approach, arguing that both the scope and the degree of moral responsibility are free from resultant luck.

In this paper, I criticize both accounts and develop an alternative. I argue, first, that Khoury’s approach leads to an implausibly far-reaching error-theory about moral responsibility. Second, Zimmerman’s account cannot account for all the ways in which moral responsibility comes in degrees. Third, these problems can be overcome by introducing a distinction between two concepts of responsibility that both come with scope and degree. The first concept I call internal responsibility, as it applies exclusively to agent-internal factors. The second concept I call external responsibility, as it applies to (partly) agent-external factors such as actions and their outcomes. Given this distinction, we can avoid the problems of Khoury’s as well as Zimmerman’s accounts while preserving the central intuition behind the rejection of resultant moral luck.

## Introduction

Consider the following well-known example of (alleged) resultant moral luck:

**Marry and Harry**

Marry–a top assassin–plans to murder Fred. She places her sniper rifle in a well-situated building, waits for Fred to come by, eventually takes careful aim and shoots. Fred is hit and dies immediately.

In another universe, Harry–also a top assassin–plans to murder Fred. He places his sniper rifle in a well-situated building, waits for Fred to come by, eventually takes careful aim and shoots. However, a bird flies in the path of Harry’s bullet, stopping it half-way. Fred is not hit and goes about his day.

Marry and Harry, we can assume, are on a par with respect to all of the factors that are typically deemed to be morally relevant: They share the same ill intentions and—in a way or up to some point—also act in the same way. Neither of them is coerced or manipulated. Both of them know what they are doing and could refrain from doing it. In fact, the only apparent difference between Harry and Marry is due to a factor that is often deemed to be morally irrelevant: namely, chance.

Still, many people have the intuition that there is a moral difference between Marry and Harry. After all, Marry committed murder, whereas Harry merely attempted to commit murder. In Marry’s case, there is a morally bad outcome, whereas in Harry’s case, there is no morally bad outcome (or at least—if we count the poor bird’s death—none that is *as* bad). Shouldn’t that lead us to a differing moral evaluation of the two cases?

The correct but trivial answer is of course: it depends. First, it depends on the general kind of moral evaluation we are interested in. Michael Zimmerman (2006) distinguishes three kinds of moral evaluation pertaining to agents: aretaic evaluations concern moral virtue and vice, deontic evaluations concern moral obligation, and hypological evaluations concern moral responsibility. I will here restrict myself to a discussion of hypological evaluations. That is, my main question is whether luck affects moral responsibility. As is common in the literature on resultant luck, I will not base my discussion on a general definition of moral responsibility, but rather trust that the reader has a sufficiently clear intuitive grasp of this notion. Moral responsibility can be bad, good, or neutral. Bad moral responsibility corresponds to blameworthiness, good moral responsibility corresponds to praiseworthiness. Neutral moral responsibility is the bland spot in between. My examples will be mostly concerned with blameworthiness, but I take the points I’m making to apply to moral responsibility more generally.

Second, it depends on the kind of hypological evaluation we are interested in. A standard distinction about moral responsibility is the distinction between its scope and its degree (see Zimmerman, 2002; Swenson, 2019). With respect to Marry and Harry, we therefore have to consider the following two questions:

Degree Question: Are Marry and Harry responsible to the same degree?

Scope Question: Are Marry and Harry responsible for the same things?

Michael Zimmerman (2002, 2006) and Philip Swenson (2019) argue that Marry and Harry are morally responsible to the same degree, but not for the same things. Even though Marry is responsible for Fred’s death while Harry is not, they are equally blameworthy. This is a partial denial of resultant moral luck: resultant luck cannot affect degrees of moral responsibility, but it can affect the scope of moral responsibility.

Andrew Khoury (2012, 2018) takes a more resolute approach: He maintains that Marry and Harry are responsible to the same degree and for the same things. Consequently, on his account, Marry is not responsible for Fred’s death, since Harry is not. In fact, Khoury arrives at the conclusion that we are responsible only for agent-internal factors such as our inner willings. This is a full denial of resultant moral luck: neither degree nor scope can be affected by resultant luck.

In this paper, I criticize both accounts and develop an alternative. As I will argue, Khoury’s position is committed to an implausible error theory concerning responsibility judgements, as it implies that we are never responsible for our actions. Error-theory is unacceptable because it is uncharitable and it ignores certain important functions of the idea that agents can be responsible for outcomes. Khoury tries to avoid the most radical form of error-theory via an identity-based argument for responsibility for actions: He maintains that we are responsible for typical actions because we are responsible for inner willings, and, given broad action individuation, typical actions can be identified with inner willings. However, this identity-based argument fails. The reason is that, as I will argue, responsibility ascriptions are intensional. That is, it is not the case that co-extensive action-descriptions can always be replaced salva veritate in the context ‘A is responsible for Φ-ing’. For this reason, Khoury’s identity-based argument is not valid.

Zimmerman and Swenson run into other problems: First, I argue that their account underestimates the moral significance of moral responsibility for outcomes. Second, it fails to account for many plausible responsibility judgements. More specifically, it cannot account for degrees of responsibility for outcomes. However, as I will argue, given that Zimmerman and Swenson allow for (luck-affected) responsibility for outcomes, and given that they are right in doing so because responsibility for outcomes is morally significant, they should also be able to account for degrees of responsibility for outcomes, as well as the influence of luck on degrees of responsibility for outcomes.

In order to avoid these problems, I propose a distinction between two concepts of responsibility that both come with scope and degree. The first concept of responsibility—internal responsibility—applies exclusively to willings. Resultant luck does neither affect the scope nor the degree of this concept. The second concept of responsibility—external responsibility—exclusively applies to actions and their outcomes. Luck affects both the scope and the degree of this concept. I argue that the distinction between internal responsibility and external responsibility solves most of the problems of Khoury’s and Zimmerman’s and Swenson’s accounts: It allows us to avoid error theory concerning responsibility judgements, it preserves the driving idea behind the rejection of resultant moral luck, and it can account for degrees of responsibility for outcomes.

Here is how I will proceed: Sect. 2 introduces two distinctions pertaining to resultant moral luck: the distinction between scope resultant luck and degree resultant luck; and the distinction between action resultant luck and outcome resultant luck. Section 3 critically discusses Khoury’s approach and argues that it cannot avoid an implausibly far-reaching error theory about ordinary responsibility judgements. Section 4 examines Zimmerman’s and Swenson’s approach and confronts it with a problem concerning the scope of responsibility for outcomes. Section 5 introduces the distinction between internal responsibility and external responsibility and discusses its application to resultant moral luck.

## Resultant moral luck: two distinctions

In this section, I work out some relevant general features of cases of resultant moral luck and introduce two distinctions pertaining to resultant moral luck. The first distinction is between scope luck and degree luck, and the second distinction is between action luck and outcome luck.

One can distinguish resultant luck and resultant moral luck. Resultant luck occurs whenever aspects of an agent’s relation to the outcomes of their behaviour are not under the agent’s control. The existence of resultant luck is uncontroversial and unavoidable. We cannot fully control what outcomes our behaviour result in, nor how it results in the outcomes it results in. Resultant *moral* luck occurs whenever aspects of an agent’s moral responsibility are affected by resultant luck. The case of Marry and Harry is a very simple case of (alleged) resultant moral luck: Marry and Harry (allegedly) differ in their moral responsibility because Marry’s behaviour causes an outcome that does not occur in Harry’s case. This crucial difference in their relation to the outcome between Marry and Harry is not controlled by either of them. So, Marry and Harry are subject to resultant moral luck.

This simple case illustrates at least two important general features of cases of resultant moral luck: First, resultant moral luck is in a certain sense contrastive: There is a *positive case*, in which the agent is morally responsible, and a *negative case*, in which a corresponding agent is *not* morally responsible. If the agent is blameworthy, they have bad luck in the positive case and good luck in the negative case. If they are praiseworthy, it is the other way around. In general, lucky agents have unlucky counterparts, and the other way around. For every unlucky Marry there’s a (possible) lucky Harry.

Second, the agent in the positive case and the agent in the negative case stand in a relation of agential equivalence. Roughly, this means that they are on a par with respect to their intentions, their relevant beliefs, the relevant factors they control and the relevant intrinsic abilities they have. Marry and Harry share the same ill intentions, they both believe that pulling the trigger will result in Fred’s death, and they both can pull the trigger or freely refrain from pulling the trigger. Since they are in this sense agentially equivalent, any difference between Marry and Harry is beyond either agent’s control.^1^

Let us now turn to the two distinctions: Scope luck occurs whenever an agent’s scope of moral responsibility is subject to resultant luck. *Degree luck* occurs whenever an agent’s degree of moral responsibility is subject to resultant luck. Marry and Harry are subject to scope luck insofar as they are responsible for different things. They are subject to degree luck insofar as they are responsible to varying degrees. As explained in the previous section, Zimmerman (2002) and Swenson (2019) deny degree luck but accept scope luck, whereas Khoury (2018) denies both degree luck and scope luck.

Scope luck can come in two varieties, since there are two ways in which the difference in outcomes of Marry’s and Harry’s actions might affect the scope of their responsibility. First, obviously, Marry and Harry might be responsible for different outcomes: Marry is responsible for Fred’s death and Harry is not. Second, Marry and Harry might be responsible for different actions: Marry is responsible for killing Fred and Harry is not. Let us say that an agent is subject to *action luck* whenever resultant luck affects what actions they are responsible for, and that an agent is subject to *outcome luck* whenever resultant luck affects what outcomes they are responsible for.^2^

Of course, action luck and outcome luck are closely related. Marry seems to be subject to action luck partly because of the *outcome* of her action: If Fred is not dead, Marry didn’t *kill* him! And she seems to be subject to outcome luck partly because her *action* resulted in the outcome: If Fred dies of an unrelated heart attack, *Marry* didn’t kill him! In a way, action luck and outcome luck are two sides of the same coin. They seem to stand or fall together. However, as we will see, the two varieties can come apart.

## Khoury’s scope internalism

Call the view that agents are responsible only for aspects of their mental lives, but never for agent-external events, *scope internalism*. Khoury (2018) defends scope internalism on the basis of the intuition that there is no resultant moral luck: According to this anti-luck intuition, Marry and Harry are in any relevant sense equally responsible. According to Khoury, this implies that they are responsible for the same outcomes. Since Harry is not responsible for Fred’s death, neither is Marry.

Khoury motivates his position in part by drawing on a brain-in-a-vat scenario that aggravates the problem of resultant moral luck. Consider Larry:

**Larry**

Larry–another top assassin–plans to murder Fred. He (justifiably but falsely) believes that he places his sniper rifle in a well-situated building, waits for Fred to come by, takes careful aim and shoots. Unbeknownst to Larry, he is a recently envatted brain-in-a-vat. In fact, he does not place his sniper rifle in a well-situated building, does not take careful aim and does not shoot, since his surroundings are not real but perfectly simulated. So, even though Larry justifiably believes that he killed Fred, he in fact did not—as Fred is still alive and well in the real world.

Leaving aside possible externalist worries, let us grant that Larry stands in the relevant agential equivalence relation to Marry. That is, Marry and Larry are on a par with respect to their intentions, their beliefs, the factors they control and the intrinsic abilities they have. At first sight, Marry and Larry then provide us with another example of outcome luck. Marry is responsible for Fred’s death while Larry is not. As Khoury emphasizes, we can extend this reasoning to the movement of their bodies: Marry is responsible for the movement of her finger, but Larry is not—as he does not even have a finger! If his finger does not move, how could he be responsible for the movement of his finger?

However, Khoury argues that we should not only hold Marry and Larry to be responsible to the same degree, but also for the same things. Since Larry is not responsible for Fred’s death and not responsible for the movement of his finger, neither is Marry.^3^ The only things for which Marry and Larry can be responsible, then, are things they share. And the only things they share are aspects of their mental lives. Khoury concludes that even Marry is responsible only for aspects of her mental life.

According to Khoury’s version of scope internalism, Marry, Harry and Larry are responsible for their *willings*. Willings are characterized ostensively, as “the mental component of action” (Khoury, 2018: 1364). They are those agential mental states that are shared between Marry, Harry and Larry. This characterization of willings is relatively neutral with respect to the nature of willings. In particular, it is not based on any overly controversial assumptions in action theory.

Why not say that Marry and Larry are responsible to the same degree, but for different things? Khoury’s objection to Zimmerman’s alternative is that this view renders the scope of responsibility entirely irrelevant. As Zimmerman (2002, p. 568) writes, “degree of responsibility counts for everything, scope for nothing”. However, Khoury argues, scope is relevant in at least two respects:

First, scope is required to determine the *quality* of an agent’s moral responsibility. There is a moral difference between an agent who negligently runs a stop sign and an agent who recklessly scares a cyclist. As Khoury points out, this difference need not be one in degree of moral responsibility: the two agents may well be blameworthy to the same degree. However, they are blameworthy in different *ways*—their responsibility has a different *quality*. This is best explained by the fact that the two agents are responsible *for* different things. One agent is responsible for negligence, the other one for recklessness. So, scope is relevant at least for the quality of an agent’s responsibility. Given this, it can also be easily explained why Marry, Harry and Larry are responsible *in the same way*: They are responsible in the same way because they are responsible for the same things, namely their willings.

Second, Khoury argues that agents are responsible for a thing only insofar as they are responsible *in virtue of* that very thing (Khoury, 2012, p. 195; Khoury, 2018, p. 1363). In other words, the objects of moral responsibility should also be grounds of moral responsibility. Call this principle the Scope-Grounding-Principle:

**The scope-grounding principle**

If an agent A is responsible for X, then X partially grounds A’s responsibility.

Zimmerman’s position violates the Scope-Grounding-Principle: It has the consequence that Marry is responsible *for* Fred’s death but not *in virtue of* Fred’s death. The fact that Marry is responsible for Fred’s death is entirely irrelevant for her degree and her quality of moral responsibility. But, Khoury asks,

If, in light of the rejection of moral luck, we can retain the possibility of responsibility for external objects only at the cost of making such responsibility empty, why bother? (Khoury, 2018, p. 1363)

Indeed, it seems pointless to hold on to a concept that has no function whatsoever. And, given that the scope of responsibility is, according to Zimmerman, irrelevant for determining both its quality and its degree, it is hard to see what functional role is left for the scope of responsibility. In contrast, Khoury’s account assigns a clear functional role to the scope of moral responsibility: The objects in the scope of moral responsibility partly ground the quality and the degree of moral responsibility. Since degree and quality of moral responsibility should be unaffected by resultant luck, the scope of responsibility should be restricted to agent-internal events.

On the face of it, however, Khoury’s scope internalism implies a quite far-reaching error-theory about ordinary responsibility judgements. According to scope internalism, it is false that Marry is responsible for Fred’s death. What is more, scope internalism apparently also implies that it is false that Marry is responsible for killing Fred or even for moving her finger. After all, *Larry* is not responsible for killing Fred or for moving his finger. And since Marry and Larry are responsible for the same things, neither is Marry responsible for killing Fred or even for moving her finger. In general, it looks like scope internalism leaves barely any ordinary responsibility ascriptions intact: according to scope internalism, no one is ever responsible for the outcomes of their actions–nor even for the actions themselves.

I consider this to be a high cost of the theory. Here is why:

First, error-theory about ordinary responsibility judgements is uncharitable. It ascribes massive error to ordinary folks. However, according to the principle of charity, we should avoid ascribing such massive error when interpreting other people—at least unless other theoretical options are thoroughly excluded.^4^ But, as I will argue later, there are plausible theoretical alternatives that avoid ascribing massive error.

Two further points concern the function of the notion that agents can be responsible for the outcomes of their actions. In his criticism of Zimmerman, Khoury assumes that the scope of responsibility should not “count for nothing”, but instead be relevant for determining the quality and the degree of moral responsibility. However, irrelevance for determining scope and degree of moral responsibility does not imply irrelevance *tout court*. Indeed, I think that the notion that agents are morally responsible for outcomes has functions that go beyond grounding their quality and degree of moral responsibility. And these functions are lost if we restrict the scope of moral responsibility to willings.

So, the second point is that moral responsibility for outcomes is relevant for determining obligations that agents acquire as a consequence of their actions. To see this, assume that Larry and Marry act simultaneously. While Larry—trapped in the simulation—justifiably but falsely believes that he kills Fred, Marry in fact successfully kills Fred in the real world. It is only natural to say that, in this situation, notwithstanding the same ill will towards Fred, only Marry is responsible for Fred’s death while Larry is not. According to Khoury, however, neither Marry nor Larry are responsible for Fred’s death. That is, Marry and Larry are morally on a par with regards to Fred’s death.

However, even granting that Marry and Larry are ultimately equally blameworthy due to their ill will, there are some important moral differences due to their different moral relations to Fred’s death. Marry has acquired *obligations* that Larry lacks. While Larry—after having been realigned with his body and having been informed what went down— may have an obligation to try to get himself convicted of attempted murder, he definitely does not have an obligation to turn himself in for the murder of Fred. After all, he actually is not Fred’s murderer. Marry, on the other hand, definitely has an obligation to turn herself in for the murder of Fred. Relatedly, when it comes to *compensation* for Fred’s death, it seems that Marry should be held accountable: She is the one who should pay for Fred’s funeral, for example.^5^

Third, we are interested in moral responsibility not only because we want to determine *how to deal with agents*, but also because we want to determine *how to deal with agent-external events*. We can roughly distinguish three kinds of (negatively-valued) agent-external events: First, there are *calamities*: negatively-valued events that are almost entirely unrelated to human agency. Examples include earthquakes or floods. Second, there are *accidents*: negatively-valued events that are caused by an agent, but not in a way that would warrant full responsibility. Examples might include some car crashes. Third, there are *bad deeds*: negatively-valued events that are intentionally and knowingly caused by an agent in a way that warrants full responsibility for the deed. Examples include deaths by murder or house fires by arson.^6^

It very much matters whether an agent-external event is a calamity, an accident, or a bad deed. Suppose Greg’s house burned down, and as a result he becomes homeless. He now has to deal with this situation. It will surely matter to Greg whether the house fire was a calamity, an accident, or a bad deed. It will matter for the question whether he is entitled to compensation. But it will also matter, for example, for his self-understanding: for whether he should see himself as a tragic figure or a crime victim. This seems to matter, then, not primarily for determining the degree of responsibility of the responsible agent, but for the question how to deal with the agent’s (external) deed.

So, moral responsibility for outcomes seems to matter not only for determining quality and degree of moral responsibility, but also for determining acquired obligations as well as establishing the distinction between calamities, accidents and bad deeds. Given that, according to Khoury’s error-theory, nobody is ever responsible for any agent-external events, these functions of moral responsibility collapse. Marry and Larry will have the same obligations, and all agent-external events will be calamities.

Now, a defender of error-theory could surely reply that these distinctions can be drawn without relying on moral responsibility for outcomes. They could, for example, introduce a relation between agents and outcomes that does not amount to moral responsibility for outcomes, but still allows us to determine acquired obligations and establish a distinction between calamities, accidents, and bad deeds. Note, however, that mere causation will not do, as mere causation does not distinguish between accidents and bad deeds, and accidentally caused outcomes do not lead to the same acquired obligations as bad deeds.^7^ I suspect that any apt agential relation to outcomes would look suspiciously similar to moral responsibility for outcomes, and it is hard to see why it should not be used to assign fulfillable truth-conditions to ordinary responsibility judgements.

Another option is to hold on to the basic idea that we are responsible only for our inner willings, while at the same time avoiding the most radical version of error-theory. Khoury takes this route by arguing that scope internalism is compatible with moral responsibility for ordinary actions such as killings. In this view, scope internalism still entails that agents are never responsible for the outcomes of their actions. But it no longer entails that agents are never responsible for their actions.

If successful, this move would indeed seem to avoid the most uncomfortable consequences of error-theory. First, though scope internalism would still ascribe massive error to ordinary folks, the error would be significantly less massive. Second, Marry and Larry could acquire different obligations because they are responsible for different actions. Third, calamities, accidents, and bad deeds could be distinguished by relying on responsibility for corresponding actions. Calamities are events that do not figure in any true action ascriptions (no one caused an earthquake), accidents are events that figure in true action ascriptions without corresponding true responsibility ascriptions (someone crashed the car, but no one is (fully) responsible for doing so), and bad deeds are events that figure in true action ascriptions with corresponding true responsibility ascriptions (someone killed Fred and is responsible for doing so).

Khoury (2018, p. 1365) argues that, given certain assumptions, scope internalism even implies that agents are responsible for ordinary actions such as killings. Applied to Marry’s case, his argument can be reconstructed as follows:

*Argument for responsibility for typical actions*

1. Marry is responsible for willing to kill Fred.
2. Marry’s killing Fred is identical to her willing to kill Fred.
3. Therefore, Marry is responsible for killing Fred.

Premise (2) rests on two claims: First, willings are basic actions. Second, actions are broadly individuated. I will briefly explain both claims.

Willings as basic actions: Marry killed Fred *by* pulling the trigger. Her pulling the trigger is *more basic* than her killing Fred. She pulled the trigger *by* moving her finger. Her moving her finger is more basic than her pulling the trigger. But is her moving her finger a *basic action*? Or is there an action *by* which she moves her finger and that is therefore more basic than her moving her finger? Khoury thinks there is: She moves her finger *by* willing to move her finger. Willings are basic actions in this sense.

Broad action individuation: Khoury adopts a broad view of action individuation due to Elizabeth Anscombe (1957) and Donald Davidson (1971). According to this view, ‘Marry’s pulling the trigger’ and ‘Marry’s killing Fred’ are two different descriptions of the same action. There is just one thing that Marry does, and *it* can be described in various ways. An especially important way to describe actions is by reference to their effects. For example, suppose that, without Marry’s knowledge, Marry’s shot causes the neighbour to have a heart attack. Then, another description of what Marry does is ‘Marry’s causing the neighbour’s heart attack’.

Combining these two theses, Khoury holds that ‘Marry’s willing to kill Fred’ is another description of what she does. It refers to the same event as ‘Marry’s moving her finger’, ‘Marry’s killing Fred’ and ‘Marry’s causing the neighbour’s heart attack’. The referent of these descriptions is a mental state, namely a willing.

An implicit background assumption of the argument for responsibility for typical actions is that responsibility ascriptions are extensional. That is, the argument is valid only if we can always replace co-referential action descriptions salva veritate in the context ‘A is responsible for Φ-ing’. As I will argue, this background assumption is false. Given broad action individuation, responsibility ascriptions are intensional.

First, a simple appeal to intuition: It just seems false to say that Marry is responsible for causing the neighbour’s heart attack, given that she doesn’t even know about the neighbour’s existence.^8^ However, Khoury clearly is committed to this counter-intuitive claim: If ‘Marry’s causing the neighbour’s heart attack’ is just another description for her willing to kill Fred, and she is responsible for willing to kill Fred, and responsibility ascriptions are extensional, then Marry is also responsible for causing the neighbour’s heart attack.

Of course, one could simply deny the intuition. Or else, one could give a pragmatic explanation of the intuition: ‘Marry is responsible for causing the neighbour’s heart attack’ might be true but infelicitous. There are moves to make here, and an appeal to intuition only gets us so far. So, what else can be said in favour of intensionality?

Second, responsibility is a member of a family of interrelated concepts that are widely held to be intensional. Responsibility is related to intentionality. What agents are responsible for depends, in part, on what they do intentionally. Marry kills Fred intentionally, but she does not cause the neighbour’s heart attack intentionally. ‘A Φs intentionally’ is an intensional context.^9^ Responsibility is also related to belief. What agents are responsible for depends, in part, on what they believe. Marry believes that she is killing Fred, but she does not believe that she is causing the neighbour’s heart attack. ‘A believes that A Φs’ is an intensional context. What is more, as Khoury emphasizes, responsibility is related to willings. What agents are responsible for depends, in part, on what they willed to do. Marry willed to kill Fred, but she did not will to cause the neighbour’s heart attack. ‘A willed to Φ’ is an intensional context. This strongly suggests that ‘A is responsible for Φ-ing’ is an intensional context as well: Marry is responsible for killing Fred, but she is not responsible for causing the neighbour’s heart attack. After all, she did not cause the neighbour’s heart attack *intentionally*, she did not *believe* that her shooting would cause the neighbour’s heart attack, and she did not *will* to cause the neighbour’s heart attack.

One might object that the mentioned clearly intensional concepts all directly ascribe particular attitudes to agents, and that this explains their intensionality. Responsibility, on the other hand, does not come with such a direct ascription of attitudes to agents. So, the motivation for treating ‘A Φs intentionally’ ‘A believes that A Φs’, and ‘A wills to Φ’ as intensional contexts does not carry over to responsibility.^10^ However, even though ‘Marry is responsible for causing the neighbour’s heart attack’ might not directly ascribe any particular attitudes to Marry, it still implies or at least strongly suggests the presence of such attitudes. If Marry is responsible for causing the neighbour’s heart attack, it is plausible to infer that she intentionally did so, or that she at least believed that her action might result in the neighbour’s heart attack. In Khoury’s picture, such inferences are not allowed. In general, the content of the responsibility-related attitudes can be completely unrelated to the actions for which the agent is responsible. Marry, for example, is responsible for causing the neighbour’s heart attack because she willed to kill Fred, and despite her complete ignorance of the neighbour’s presence. In a way, singling out responsibility as an extensional concept in an otherwise intensional family of concepts comes at the cost of cutting the family ties.

Perhaps, one might still insist on the extensionality of responsibility and try to explain all contrary appearances away. This brings us to another problem: Moral responsibility for actions in the extensional sense does still not lead to an acceptable version of scope internalism. The fact that Marry is responsible for causing the neighbour’s heart attack should not lead us to ascribe obligations to Marry with respect to the neighbour’s heart attack. So, responsibility in the extensional sense is not relevant for acquired obligations. The fact that Marry is responsible for causing the neighbour’s heart attack should not lead us to say that the neighbour’s heart attack is Marry’s bad deed rather than an accident. So, responsibility in the extensional sense does not allow us to draw the distinction between accidents and bad deeds. Generally, responsibility in the extensional sense is not of much help when it comes to avoiding the uncomfortable consequences of error-theoretical scope internalism.

What is more, note that—at least superficially—the argument for responsibility for typical actions reintroduces one form of scope luck, namely action luck. Given the extensionality of responsibility, Marry is responsible for killing Fred. But Larry is still not responsible for killing Fred. Therefore, Marry and Larry are responsible for different actions.

Khoury might reply that this form of action luck is harmless precisely because responsibility is extensional. When we are saying that Marry is responsible for causing the neighbour’s heart attack, we are really saying nothing more than that she is responsible for her willing to kill Fred—we just do so in a very confusing way. Specifically, it is not the case that Marry and Larry are responsible for different things: they both are responsible only for willing to kill Fred. It is just that this fact can be described in various ways, and that Marry’s situation allows for descriptions that Larry’s situation does not allow for. Action luck, then, is merely a matter of description and therefore harmless.^11^

This reply, I think, is not entirely satisfying: It rests on the idea that, even though ‘Marry is responsible for killing Fred’ is true, this statement is just a confusing way of saying that she is responsible for her willing. It does not add any significant content to the claim that she is responsible for her willing. But then, again, it is hard to see how the truth of such statements could do any theoretical work, or be of any help in avoiding the uncomfortable consequences of error-theoretical scope internalism.

In sum, I conclude that Khoury’s attempt to avoid the most radical version of error-theoretical scope internalism via an identity-based argument for responsibility for typical actions fails. Consequently, scope internalism does have the highly counterintuitive consequence that agents are not responsible for their typical actions. This means that ordinary responsibility judgements like ‘Marry is responsible for killing Fred’ are false. This far-reaching error-theory uncharitably ascribes massive error to ordinary folks, fails to account for the obligations agents acquire due to their responsibility for outcomes, and fails to do justice to the observation that we are interested in moral responsibility not only because of our interest in blameworthy agents, but also because of our interest in the events for which agents are to blame.

## Scope luck without degree luck

Zimmerman (2002) and Swenson (2019) allow agent-external events (like Fred’s death) and typical actions (like killing Fred) to be in the scope of moral responsibility. As they see it, Marry, Harry and Larry are responsible for different actions and for different outcomes. So, they are subject both to outcome luck and to action luck. However, they take this to be, in a way, morally irrelevant. The reason is that they combine the acceptance of scope luck with a denial of degree luck: Even though Marry, Harry, and Larry are responsible for different things, they still are responsible to the same degree. Since degree is all that matters for blame- and praiseworthiness, scope luck is morally irrelevant. Zimmerman writes:

[D]o not be misled […] into thinking that I am invoking two types of moral responsibility here. On the contrary, there is just one type. George and Georg are to be evaluated in exactly the same way, even though George is to blame for something that Georg is not. They are equally responsible; if George is deserving of a particular reaction, then Georg is deserving of the very same reaction. This indicates that whether there is something for which one is responsible is immaterial; all that matters, fundamentally, is whether one is responsible. Degree of responsibility counts for everything, scope counts for nothing, when it comes to such moral evaluations of agents. (Zimmerman, 2002, p. 568)

For our purposes, this passage can be read such that George and Georg are equivalent to Marry and Harry. Here, the idea is that we have one concept of moral responsibility that comes with scope and degree. However, the scope of this concept is detached from its degree. The fact that Marry is responsible for a death not even partly determines how responsible she is.^12^

As already briefly discussed in Sect. 3, Khoury’s main issue with this view is that it renders the scope of moral responsibility entirely irrelevant. Given its irrelevance for grounding both the degree and the quality of moral responsibility, it is unclear why we should hold on to the notion that agents are responsible for outcomes. However, one should be careful about the exact interpretation of the claim of moral irrelevance here. Zimmerman maintains that scope counts for nothing when it comes to determining degrees of moral responsibility. This is compatible with scope being relevant for other things. As I have argued above, scope indeed seems to be relevant for determining the agent’s acquired obligations. Agents who are blameworthy for an outcome acquire an obligation to make good for it. Scope also is relevant for the question how to deal with agent-external events. Events for which someone is responsible (e.g. bad deeds) warrant different reactions than events for which no one is responsible (e.g. calamities or accidents). So, Zimmerman and Swenson are right in allowing for luck-affected responsibility for typical actions and outcomes.

Once we have accepted that responsibility for typical actions and outcomes is significant, we should be ready to fully account for the phenomenon. As I will argue, however, the simple distinction between scope and degree of responsibility does not allow for a proper analysis of luck-affected responsibility for outcomes. While it allows us to assign degrees of responsibility, and also to assign responsibility for outcomes, it does not allow us to adequately assign *degrees of responsibility for outcomes*.

The idea that agents can be responsible for outcomes to varying degrees has recently sparked some discussion (see Bernstein, 2017; Kaiserman, 2021; Sartorio, 2020). Alex Kaiserman introduces this idea as follows:

An important platitude about responsibility is that it comes in degrees: someone can be more or less responsible for an outcome, depending on their degree of agential involvement in bringing the outcome about. (Kaiserman, 2021, p. 5)

Kaiserman’s degrees of responsibility are not Zimmerman’s degrees of responsibility. This is easy to see: Marry and Larry do not differ with respect to Zimmerman’s degrees of responsibility. They are equally blameworthy. However, they differ quite significantly with respect to their degrees of responsibility for Fred’s death. While Marry is responsible for Fred’s death to some (presumably high) degree, Larry is not at all responsible for this outcome. Note that, as far as degrees of responsibility *for outcomes* is concerned, Marry and Larry are actually subject to degree luck. Notwithstanding their agential equivalence, they differ with respect to their degree of responsibility *for Fred’s death*. This, of course, is not what Zimmerman and Swenson deny when they deny the existence of degree luck. This observation alone already establishes, I think, that there are two distinct notions of degrees of responsibility at play here.

In a different context, Zimmerman (1985) also speaks of degrees of responsibility for outcomes. He seems to suggest, however, that degrees of responsibility for outcomes are entirely parasitic on degrees of responsibility in Zimmerman’s (2002) sense.^13^ This is quite plausible for some scenarios. Consider Kerry, for example—another assassin who successfully kills Fred. In contrast to Marry, Kerry was somewhat coerced into killing Fred. Plausibly, the coercion lowers both her degree of responsibility in Zimmerman’s sense and her degree of responsibility for Fred’s death. Such cases might motivate the principle that, *if an agent is responsible at all for an outcome, her degree of responsibility for that outcome equals her degree of responsibility in Zimmerman’s sense*.

Other cases, however, are not as easy to handle. Consider another variant of our increasingly peculiar murder-case:

**Garry and Jerry**

Garry–a top assassin–plans to murder Fred. He places his sniper rifle in a well-situated building, waits for Fred to come by, eventually takes careful aim and shoots. Fred is hit.

On the other side of the road, Jerry–also a top assassin–plans to murder Fred. He places his sniper rifle in a well-situated building, waits for Fred to come by, eventually takes careful aim and shoots.

Unbeknownst to Jerry, the bullets in his rifle have been replaced with blanks. However, Jerry’s shot startles a passing bird, which then flies into a lamppost, dies from it, and finally falls on Fred’s head.

As it happens, Garry’s shot alone would have been almost, but not quite sufficient to kill Fred. It would have merely badly injured him. However, the bird that falls on his head—which, in the absence of Garry’s shot, would have caused but a bump—tips him over the edge. Fred dies.

It seems plausible to me that, in this situation, both Jerry and Garry are somewhat responsible for Fred’s death, but Jerry is significantly *less* responsible for Fred’s death than Garry. This is so despite the agential equivalence between Jerry and Garry (they have the same willings etc.). What we have here is a case of what Sara Bernstein calls ‘proportionality luck’, which “involves circumstances out of an agent’s control either increasing or decreasing that agent’s proportion of moral responsibility for an outcome” (Bernstein, 2017: 168).

The difference in degrees of moral responsibility for the outcome is best explained by a difference in the causal relation to the outcome. In fact, the case combines two different features of the causal relation that have been identified as responsibility-diminishing: First, the causal relation between Jerry’s shot and Fred’s death is deviant. Deviant causation diminishes responsibility (see Bernstein (2019)). Second, Jerry’s shot causally contributes less to Fred’s death than Jerry’s shot. Lesser causal contribution diminishes responsibility (see Bernstein (2017) and Kaiserman (2021)).

The idea that degrees of causal contribution are relevant to degrees of responsibility for an outcome is a natural extension of the idea that (non-graded) causation is relevant to (non-graded) responsibility for an outcome, which should be more or less uncontroversial. Marry and Larry differ with respect to their moral responsibility for Fred’s death because Marry caused Fred’s death and Larry did not. Parallelly, Garry and Jerry differ with respect to their degree of moral responsibility for Fred’s death because Jerry contributed less to Fred’s death than Garry.^14^

Given this causal factor, degrees of responsibility for outcomes and degrees of responsibility in Zimmerman’s sense can come apart. Due to their agential equivalence, Garry and Jerry are equally responsible in Zimmerman’s sense of degrees of responsibility. However, due to their different causal relation to Fred’s death, they differ in their degree of responsibility for that outcome. This shows that degrees of responsibility for outcomes are not entirely parasitic on degrees of responsibility in Zimmerman’s sense.

One might object in one of two ways: Either, one might deny that Garry and Jerry are, in fact, equally responsible in Zimmerman’s sense. The consequence would be a full re-instantiation of degree luck. I do not find this consequence very attractive, and would like to avoid it. Or, one might deny that Garry and Jerry are differentially responsible for Fred’s death. The consequence would be that causal factors play no role for degrees of responsibility for outcomes. I do not find this particularly plausible either. Luckily, both positions can be avoided by taking seriously the idea that degrees of responsibility in Zimmerman’s sense and degrees of responsibility for outcomes are truly separate phenomena.

## Two concepts of responsibility

Here are the main lessons of the discussion up to now:

First, I think Khoury is right in emphasizing that the desire to avoid resultant moral luck requires assigning a special status to agents’ internal mental states, especially their willings.

Second, however, we should not let this desire lure us into error-theory about ordinary responsibility ascriptions. Error-theory is highly implausible: it uncharitably ascribes massive error to ordinary folks, and it overlooks important functions of the notion that we can be responsible for actions and outcomes (in addition to willings).

Third, I agree with Khoury in that there is a certain oddness to Zimmerman’s claim that agents are responsible for all kinds of outcomes, while these outcomes do not play any role in determining their degree or quality of moral responsibility. If we can avoid separating the scope of moral responsibility from its grounds in this way, we should do so.

Fourth, however, Zimmerman and Swenson still take a step in the right direction. Their position allows us to shield an agent’s moral core—their degree of blame-and praiseworthiness in Zimmerman’s sense—from the influence of resultant luck, while at the same time to avoid error-theory.

Fifth, there is an interesting thread in the recent debate about resultant moral luck—and especially its relation to causation—in which the idea that agents can be responsible for outcomes to varying degrees plays an important role. The notion of degrees of responsibility for outcomes here is not the same as Zimmerman’s notion of degrees of responsibility. It would be nice if we could reconcile the insights from this debate about the relation between causation and responsibility for outcomes with a denial of moral degree luck in Zimmerman’s sense.

I suggest that we account for these observations by introducing two concepts of responsibility. One concept exclusively applies to agent-internal events. Therefore, I call it internal responsibility. The other concept applies to typical actions and outcomes—that is, to (partly) agent-external events. Accordingly, I call it external responsibility.^15^ By introducing these two concepts, I hope to capture both the most plausible aspects of Khoury’s scope internalism and the most plausible aspects of Zimmerman’s denial of degree luck and embracement of scope luck, while avoiding all the problems.

Let us start with internal responsibility. It seems right to say that, *in some sense*, Marry, Harry, Larry, Garry and Jerry all show the same kind of behaviour. All *they* really do is will to kill—the rest is up to nature. This common element should be the object of internal responsibility: Agents are internally responsible only for agent-internal events—willings in Khoury’s sense. Accordingly, internal responsibility is not subject to scope luck. That is, resultant luck does not affect the scope of internal responsibility.

Internal responsibility plausibly comes in degrees. As stated in Sect. 4, Marry is more responsible for killing Fred than Kerry, who was somewhat coerced into killing Fred. Plausibly, the coercion also leads to differences in their responsibility for *willing* to kill Fred. Murderous Marry is more responsible for her willing than coerced Kerry. However, this difference in degrees of internal responsibility is not due to resultant luck.^16^ The outcomes of an agent’s willing do not even partly ground the degree to which an agent is internally responsible *for the willing*. This, I take it, is the kind of degrees of responsibility that Zimmerman (2002) is concerned with.

So, with respect to internal responsibility, it is most plausible to reject both scope luck and degree luck.

However, this is not yet the full story. There is also external responsibility. This concept applies to our actions and their outcomes. Marry is responsible for killing Fred and for Fred’s death. Harry and Larry are not. This is due to the difference in their causal relations to Fred’s death: While Marry’s action causes Fred’s death, Harry’s and Larry’s action do not. As this is a factor that is not in either agent’s control, the scope of external responsibility *is* affected by scope luck.

External responsibility also comes in degrees. Jerry is less responsible for Fred’s death than Garry. This, again, is due to the difference in their causal relations to Fred’s death: While Jerry’s causal contribution to Fred’s death is deviant and small, Garry’s causal contribution is non-deviant and big. As this is a factor that is not in either agent’s control, degrees of external responsibility are subject to degree luck. This is the kind of degrees of responsibility that Bernstein (2017) and Kaiserman (2021) are concerned with.

So, with respect to external responsibility, it is most plausible to accept both scope luck and degree luck.

What is the significance of the two kinds of moral responsibility? Where does the distinction leave us with respect to the important question *how to treat* the various villainous figures of our examples?

As I see it, internal responsibility is in a way the more central notion. It is the notion that is most plausibly tied to the moral reactive attitudes (see Strawson, 1962).^17^ An ideal observer who securely knows about Larry’s, Garry’s and Jerry’s ill will should resent them as much as Marry. All our villains deserve the same kind and amount of resentment. I am inclined to think that they also ultimately deserve the same kind and amount of punishment.^18^ Thomson (1989, p. 207) asks whether morally unlucky agents should be thrown “into a deeper circle of hell” than their lucky counterparts. I do not think they should. If any of them deserves hell, they all deserve the same kind of hell.^19^ This is the main intuition behind our uneasiness with resultant moral luck, and it is a fair one.^20^

However, as I have argued, our interest in deserved resentment and deserved punishment does not exhaust our interest in moral responsibility. There are reasons why we are interested in who is responsible for some agent-external event that go beyond the issues of how much resentment and punishment agents deserve. First, we want to know who has an obligation to make good for unwelcome events. Since Marry is responsible for Fred’s death, she acquires obligations with respect to that outcome. Larry does not acquire corresponding obligations. Second, we are interested in whether unwelcome events are calamities, accidents, or bad deeds. Since someone is responsible for Fred’s death, it is a bad deed. This has consequences: Fred’s funeral and his eulogy, for example, would probably look a lot different if his death was an accident or a calamity.

Another question concerns blame and blameworthiness. Do our villains all deserve the same kind and amount of blame? Are they equally blameworthy? Here, the answer is less straightforward: Once we have recognized the ambiguity in moral responsibility, it is easy to see that there is a corresponding ambiguity in the terms ‘blame’ and ‘blameworthiness’. We should blame Larry just as we should blame Marry, but we should surely not blame Larry *for Fred’s death*. Marry and Larry are, in a way, equally blameworthy, but they surely are not equally blameworthy *for Fred’s death*. This strongly suggests that there is an internal and an external sense of blame and blameworthiness, corresponding to the two senses of responsibility. Consequently, the connection between moral responsibility and blameworthiness remains intact. Bad internal moral responsibility corresponds to internal blameworthiness, bad external responsibility corresponds to external blameworthiness. As argued above, internal blameworthiness most plausibly has a direct connection to deserved resentment and deserved punishment. But this does not imply that external blameworthiness is an entirely empty and superfluous notion. It is relevant for acquired obligations, and it makes the difference between accidents and bad deeds.

The resulting picture of resultant moral luck cases is then the following: There is a sense in which lucky agents and their unlucky counterparts are equally responsible both with respect to degree and with respect to scope. They are internally responsible to the same degree and for the same things. This is why lucky and unlucky agents deserve the same kind and amount of resentment and punishment. In this respect, they are morally on a par. There is another sense in which lucky agents and their unlucky counterparts are differentially responsible both with respect to scope and with respect to degree. They are externally responsible for different things and to different degrees. This is why they acquire different obligations. Resultant moral luck cases are cases of external scope- and degree-luck without internal scope- and degree-luck.

Introducing a distinction usually raises the question of relation: How are internal responsibility and external responsibility connected to one another?

Marry is externally responsible for killing Fred and for Fred’s death partly *because* she is internally responsible for willing to kill Fred. External responsibility is partly grounded in internal responsibility. But it is not the case that Marry is internally responsible for willing to kill Fred partly because she is responsible for Fred’s death. Internal responsibility is not even partly grounded in external responsibility. This grounding-asymmetry marks another sense in which internal responsibility is more central.

External responsibility is grounded in internal responsibility because it is, as it were, *anchored* in internal responsibility: The underlying idea is that we are *primarily* internally responsible for our willings. This makes us externally responsible for certain actions and outcomes that are properly connected to those willings. One main challenge for a more thorough explication of external responsibility is to specify and study the relevant sort of connection. After all, we are not responsible for all events that are *causally* connected to the willings for which we are responsible. For example, we are obviously not responsible for distant causal consequences of our willings that we had no way of foreseeing. What is more, as the case of Garry and Jerry shows, there are intricate relations between certain more specific features of the agents’ causal connection to the outcome on the one hand and their degrees of external responsibility on the other. Causal deviance and low degrees of causation diminish degrees of external responsibility. These observations deserve further attention.

If this is right, an account of external responsibility should build on an account of internal responsibility. It is then clear that we have two distinct but related concepts: Internal responsibility gives us the conditions under which agents are responsible for their inner willings. External responsibility specifies a connection between those willings for which we are internally responsible and those actions and outcomes for which we are externally responsible.

Let us return to the five lessons I outlined in the beginning of this section.

First, what about Khoury’s point that a thorough rejection of resultant moral luck requires assigning a special status to willings? This insight is captured by the fact that internal responsibility indeed takes willings as its objects, and that it is the more central notion in that it warrants resentment as well as punishment and grounds external responsibility. Willings, then, are absolutely crucial to our moral practice, and this is the reason why our most significant moral features are shielded from resultant luck.

Second, the centrality of willings does not lure us into error-theory about ordinary responsibility judgements. When we say that Marry is responsible for killing Fred or for Fred’s death, we are not saying something *false*. It is just that we are not saying something directly *about internal responsibility*. The truth conditions of ordinary responsibility judgements concerning actions and outcomes are to be given in terms of external responsibility. We are not committed to uncharitably ascribing massive error to ordinary folks, and we can account for the important functions of external responsibility.

Third, we can avoid separating the scope of moral responsibility from its grounds. The Scope-Grounding-Principle holds for internal and for external responsibility. The objects of internal responsibility—willings—also ground the quality and the degree of internal responsibility. The same is true for external responsibility: The objects of external responsibility—actions and their outcomes—are also grounds of external responsibility. When an agent is responsible for something, they are also responsible in virtue of that something.

Fourth, we can go beyond the important step in the right direction that Zimmerman’s account makes. While Zimmerman’s account allows us to ascribe moral responsibility for outcomes, it does not allows us to do full justice to the observation that responsibility for outcomes comes in degrees. The view defended here recognizes that degrees of responsibility for outcomes and degrees of responsibility in Zimmerman’s sense can come apart, and that degrees of responsibility for outcomes are a separate phenomenon that deserves further scrutiny.

Fifth, this enables a more detailed analysis of the influence of causation on degrees of responsibility for outcomes, and especially allows us to account for the observation that causal deviance and degrees of causation are relevant to degrees of responsibility for outcomes. In other words, we can reconcile the interesting recent work on the connection between causation and degrees of responsibility for outcomes (Bernstein, 2017; Kaiserman, 2021) with a far-reaching denial of resultant moral luck.

## Conclusion

Let us take stock: In Sect. 2, I have introduced some general features of resultant moral luck and argued that we should distinguish degree luck from scope luck and action luck from outcome luck. In Sect. 3, I have introduced Khoury’s scope internalism and argued that it cannot avoid an implausibly strong error-theory about ordinary responsibility judgements. In Sect. 4, I have discussed Zimmerman’s and Swenson’s denial of degree luck and embracement of scope luck, and have argued that it fails to do justice to degrees of responsibility for outcomes. Finally, in Sect. 5, I have distinguished between internal responsibility and external responsibility and discussed how this solves the problems of Khoury’s and Zimmerman’s accounts of moral luck.

I conclude that a plausible general theory of moral responsibility should take both internal and external responsibility into account. Focussing exclusively on external responsibility renders it impossible to adequately capture the intuition that, in a very important sense, there is no resultant moral luck. An exclusive focus on internal responsibility, on the other hand, would leave out one of the core applications of moral responsibility: actions and their outcomes. It would thereby lead to an implausible error-theory and fail to do justice to the functions of moral responsibility for outcomes. Consequently, the distinction is required to shed light on the whole range of responsibility judgements and resultant moral luck cases.

## Footnotes

1 As Carolina Sartorio (2012) points out, the simple case of Marry and Harry also has some features that are not essential to cases of resultant moral luck. First, it is not essential to cases of resultant moral luck that an outcome occurs in the positive case that does not occur in the negative case. It might just as well be the case that the outcome occurs in both cases, but the agents are differently related to the outcome. Second, it is not essential to cases of resultant moral luck that the agents positively act rather than omit to act. Here, I will mostly restrict my discussion to ‘typical’ cases of resultant moral luck.

2 Note that I explicitly introduce both distinctions with respect to resultant luck. The same distinctions could also be drawn with respect to other forms of moral luck (i.e. circumstantial, constitutive and causal moral luck, see Nagel 1993). That is, there could for example be scope circumstantial luck and degree circumstantial luck, where scope circumstantial luck occurs whenever an agent’s scope of moral responsibility is subject to *circumstantial* luck and degree circumstantial luck occurs whenever an agent’s degree of moral responsibility is subject to *circumstantial* luck. Accordingly, it would be more precise to throughout talk of scope resultant moral luck, degree resultant moral luck, action resultant moral luck and outcome resultant moral luck. However, this would be much less convenient, and as this paper exclusively addresses resultant moral luck, I will go with the shorter expressions.

3 Note that there are two ways in which one might resist the conclusion that Marry, Harry and Larry are subject to scope luck. The sane way is the one Khoury takes: Neither of them is responsible for such things as Fred’s death or the movement of a finger. The crazy alternative would be to claim that all of them are responsible for these things. That is, Harry is responsible for Fred’s death even though Fred is still alive, and Larry is responsible for the movement of his finger even though his finger does not move. I have a hunch that the crazy alternative is less crazy than it at first seems—or, at least, that the extent of its craziness does not far outreach the extent of the craziness of some extant positions. After all, deniers of *circumstantial* moral luck are already committed to the idea that we are sometimes responsible in virtue of merely possible actions and intentions (cf. Zimmerman (2002) and Hanna (2014) for critical discussion). But if we are already, in a way, morally modal beings, why not say that we are responsible for (and in virtue of) merely possible *outcomes* of our actions?

4 See for example Wright (1994) for an argument against error-theory that is based on the principle of charity, and Daly and Liggins (2010) for critical discussion.

5 Swenson (2019) makes a similar point framed in terms of liability.

6 Parallelly, we could distinguish three kinds of positively-valued events: godsends, flukes, and good deeds.

7 See also Swenson (2019) for an argument to that effect.

8 If you think that some kind of negligence is involved here, assume that it was very important to Marry not to hurt or disturb anyone else, that she took drastic measures to ensure that nobody would hear her, and that still – due to some unforeseeable circumstances and through no fault of her own – her action caused the neighbour’s heart attack.

9 See Davidson (1967) and Antony (1987).

10 Thanks to an anonymous reviewer for raising this worry.

11 Thanks to an anonymous reviewer for suggesting this line of thought.

12 Zimmerman (2002) also defends a controversial theory about other forms of moral luck. More specifically, in reaction to circumstantial moral luck, he defends the idea that agents can be responsible to a high degree without being responsible for anything. As I am here exclusively concerned with resultant moral luck, I will not discuss this suggestion.

13 Zimmerman (1985, p.117) approvingly speaks of “the degree to which A is responsible for the outcome” and “diminished responsibility for the outcome”, but suggests that “this might simply be a reflection of the fact that A is more to blame than B”.

14 Zimmerman (1985: 117) briefly considers but quickly dismisses this idea, since he holds that causation is “a factor which admits of no degrees”. However, recently various authors defend the idea that causation admits of degrees and spell out interesting notions of degrees of causation (e.g. Chockler and Halpern (2004), Kaiserman (2016), Demirtas (2022)). In my opinion, Zimmerman is a bit too quick in dismissing this idea. See also Kaiserman (2021) for discussion of Zimmerman’s (1985) position.

15 Note that the term ‘external responsibility’ is not meant to imply that responsibility in this sense is a *purely* external matter. On the contrary, as I will briefly argue later, external responsibility in this sense is anchored in internal responsibility.

16 However, this is compatible with the view that degrees of internal responsibility are affected by other forms of moral luck. For example, Hanna’s (2014) defence of circumstantial moral luck plausibly implies that degrees of internal responsibility are affected by circumstantial luck. Relatedly, Zimmerman (2002, p. 575) allows for a specific kind of constitutive luck involving an agent’s essential properties to affect degrees of responsibility.

17 It is my understanding that most extant theories of moral responsibility, in one way or another, focus on the connection between moral responsibility and the moral reactive attitudes such as resentment. Strawson’s (1962) influential reactive attitudes account can be seen as an attempt to explain moral responsibility in terms of reactive attitudes. Other accounts (e.g. Fischer and Ravizza 1998) instead focus on the conditions that an agent must fulfil in order to be an appropriate target of reactive attitudes. I think that the primary target of such theories is internal responsibility, as internal responsibility is most plausibly associated with reactive attitudes. At the same time, I think that such theories are incomplete as long as they are not amended by a notion of external responsibility that accounts for the intricate relation between causation and responsibility for outcomes.

18 That is not to say that the law should treat murder and attempted murder alike. Judges are not ideal observers, and Thomson’s question whether our villains should be thrown in the same circle of hell is different from the question whether they should be thrown in jail for the same amount of time.

19 If we think of the devil as a torturer, though, I think that nobody deserves hell.

20 An anonymous reviewer points out the possibility of distinguishing a weak anti-luck intuition, according to which there is an important sense in which lucky agents and their unlucky counterparts are equally responsible, from a strong anti-luck intuition, according to which there is no sense in which they are differentially responsible. Given this characterization, I here merely account for the weak anti-luck intuition, as I allow for a sense in which lucky and unlucky agents are differentially responsible. However, given the significance of the sense in which lucky and unlucky agents are equally responsible, I still think that this denial of resultant moral luck captures the main intuition behind our uneasiness with resultant moral luck.
